# Therapeutic Effects of Platelet-Derived Extracellular Vesicles in a Bioengineered Tendon Disease Model

**DOI:** 10.3390/ijms23062948

**Published:** 2022-03-09

**Authors:** Ana L. Graça, Rui M. A. Domingues, Isabel Calejo, Manuel Gómez-Florit, Manuela E. Gomes

**Affiliations:** 1 3B’s Research Group, I3Bs—Research Institute on Biomaterials, Biodegradables and Biomimetics, University of Minho, Headquarters of the European Institute of Excellence on Tissue Engineering and Regenerative Medicine, AvePark, Parque de Ciência e Tecnologia, Zona Industrial da Gandra, 4805-017 Guimarães, Portugal; ana.graca@i3bs.uminho.pt (A.L.G.); rui.domingues@i3bs.uminho.pt (R.M.A.D.); isabel.calejo@i3bs.uminho.pt (I.C.); 2 ICVS/3B’s–PT Government Associate Laboratory, 4805-017 Guimarães, Portugal

**Keywords:** extracellular vesicles, platelets, tendinopathy, tendon-derived cells, in vitro models, fibers, tissue engineering

## Abstract

Tendon injuries represent over 30–50% of musculoskeletal disorders worldwide, yet the available therapies do not provide complete tendon repair/regeneration and full functionality restoring. Extracellular vesicles (EVs), membrane-enclosed nanoparticles, have emerged as the next breakthrough in tissue engineering and regenerative medicine to promote endogenous tissue regeneration. Here, we developed a 3D human in vitro model mimicking the signature of pathological tendon and used it to evaluate the influence that different platelet-derived EVs might have in tendon tissue repair mechanisms. For this, different EV populations isolated from platelets, small EVs (sEVs) and medium EVs (mEVs), were added to the culture media of human tendon-derived cells (hTDCs) cultured on isotropic nanofibrous scaffolds. The platelet-derived EVs increased the expression of tenogenic markers, promoted a healthy extracellular matrix (ECM) remodeling, and the synthesis of anti-inflammatory mediators. These findings suggest that platelet EVs provided relevant biochemical cues that potentiated a recovery of hTDCs phenotype from a diseased to a healthy state. Thus, this study opens new perspectives for the translation of platelet-derived EVs as therapeutics.

## 1. Introduction

Tendon disorders are a common clinical problem that dramatically affects the quality of life of individuals, causing pain, swelling and restricted movement [1]. From pathophysiological knowledge, tendinopathies frequently involve an unresolved inflammatory scenario that provokes hypercellularity, neovascularization, and a dysregulation of the critical balance between extracellular matrix (ECM) remodeling proteases and their inhibitors, which alters native ECM components and organization (loss of collagen anisotropic organization), resulting in reduced biomechanical strength [2]. Currently, conservative clinical approaches to treat tendon injuries involve pain management, steroidal and non-steroidal anti-inflammatory drugs, diverse rehabilitation strategies over a course of several months or years, injection with platelet-rich products, or, when these conservative approaches fail, surgery in severe cases [3,4,5,6,7,8]. Nonetheless, these strategies do not tackle the etiology of tendinopathies, increasing the risk of joint instability that may progress into early onset of severe degenerative conditions and, ultimately, in acute tendon rupture [2]. Thus, the development of more effective therapies against tendon disorders that blunt the pro-inflammatory response and promote pathology resolution by reestablishing the cellularity and ECM homeostasis of the tendon, encouraging effective tissue regeneration, are needed.

Extracellular vesicles (EVs) are cell-secreted nanosized lipid bilayer-enclosed particles, which contain biomolecules sorted and packed from cell cytosol, that regulate multiple biological functions [9], including stem cell differentiation, angiogenesis, regulation of immune response, and ECM remodeling [10,11]. These vesicles are normally classified by their size and biogenesis into small (30–150 nm) and medium (100–1000 nm) EVs, which are actively released by cells, containing different types of molecular cargo (e.g., RNAs, proteins, metabolites) that reflects their cells of origin [12,13]. Over the past few years, EV-based treatments have shown promising results in vitro and in clinical trials in the treatment of multiple diseases [14]. Moreover, EVs show lower immunogenicity than cell therapies and higher stability than growth factor-based therapeutics [15]. Nevertheless, the field has largely relied on mesenchymal stem cells-derived EVs, a source from which the production of clinically relevant quantities in physiological conditions and showing the required reproducibility is still the main limitation in the field [16].

Platelets are the first cells that accumulate at sites of injury to re-establish tissue homeostasis orchestrating the highly complex microenvironment present in wound healing by providing a wide range of biochemical signals and structural elements [17]. Actually, platelets are able to release several growth factors involved in different phases of tissues healing, such as platelet derived growth factor (PDGF), transforming growth factor-beta (TGF-β), vascular endothelial growth factor (VEGF), basic fibroblast growth factor (bFGF), or epidermal growth factor (EGF), and, theoretically, their presence must be involved in the healing and regeneration mechanisms of tendon tissues [18]. Nevertheless, systematic reviews and meta-analyses have not confirmed the significant efficacy of platelet-rich plasma in the management of tendinopathies [19,20,21]. These results might be mainly related to the lack of standardization and individual variability, but most importantly, due to the uncontrolled release of platelet biomolecules with diverse, and in some cases, undesired biological effects of some components from the platelet-rich products [17,22]. In this context, platelet EVs are a potential and underexplored source of sorted bioactive molecules, including different growth factors [23], that can overcome some of the aforementioned limitations. Platelet EVs offer unprecedented perspectives for personalized therapies, since they can be obtained from autologous sources from a simple blood extraction [8,22,24,25]. Moreover, platelet EVs can also be obtained on a large scale from otherwise discarded expired platelet units for clinical use, and have low batch-to-batch variability in terms of growth factors composition [18,26], which would be reflected in EVs with a more standardized biomolecule signature. Moreover, EVs offer not only a controlled bioactive cargo release system, but also a high selective targetability [15].

Herein, we aimed to evaluate the regenerative potential of platelet-derived EVs in a disease-like tendon model. In previous works, the scar-like architecture of diseased tendon was mimicked using isotropic (random) fibrous meshes, which profoundly affected cell behavior [27,28,29]. In this work, we relied on previously developed composite living fibers consisting of a nanofibrillar isotropic core coated with a platelet lysate (PL)-based hydrogel encapsulating human tendon-derived cells (hTDCs), which recreate microstructural cues and three-dimensional architecture of the tendon diseased microenvironment [30,31]. This model was then used to test the effects of different platelet-derived EV populations as potential biochemical cues to promote hTDCs healthy phenotype recovery. The role of platelet EVs on hTDCs was assessed by analyzing tendon markers and ECM deposition and remodeling. Building on this knowledge, and considering the translational potential of EVs, we aim to provide important insight into designing clinically feasible systems that can be used as cell-free therapies to modulate the tendinopathy microenvironment and promote a challenging regenerative tendon response.

## 2. Results and Discussion

### 2.1. Characterization of the 3D Tendon Disease In Vitro Model and Platelet EVs

Healthy tendons are mainly composed of aligned collagen fibers hierarchically organized from collagen fibrils to fiber bundles with a scarce cellular population mainly composed of tenocytes and tendon stem/progenitor cells [32]. However, when injured, tendon acquires a fibrotic phenotype during the healing phases, characterized by a loss of ECM fibers alignment and increased cellularization [33,34]. Despite the increased knowledge in tendon pathophysiology over the years, the main limitation in studying the potential of new drugs or therapeutic agents to restore tendon health is the lack of in vitro models that recapitulate the complexity of the tendon niche. Previous works relied on random 2D meshes to mimic fibrotic tissue and predispose seeded tendon cells towards inflammatory and degenerative pathways [28,29], although these lack the relevant 3D context of native tissues. 

**Figure 1:**
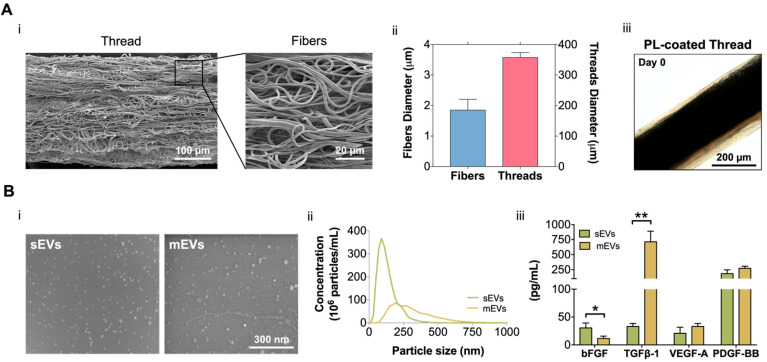
(**A**) (i) Scanning electron microscopy (SEM) micrographs of the produced misaligned fiber threads. Scale bars: 100 µm and 20 µm. (ii) Isotropic fibers and threads diameter. Data are presented as mean ± standard deviation (SD) (n = 50). (iii) Platelet lysate (PL) coating at day 0. Scale bar: 200 µm. (**B**) (i) SEM images of small and medium extracellular vesicles (sEVs; mEVs) isolated from PL. Scale bar: 300 nm. (ii) Size distribution profile of sEVs and mEVs analyzed with nanoparticle tracking analysis (NTA) (n = 3). (iii) EV basic fibroblast growth factor (bFGF), transforming growth factor-beta 1 (TGF-β1), vascular endothelial growth factor-A (VEGF-A), and platelet-derived growth factor-BB (PDGF-BB) quantification. Data are presented as mean ± SD (n = 3). Statistical significance: * *p* < 0.05, ** *p* < 0.01.

**Figure 2:**
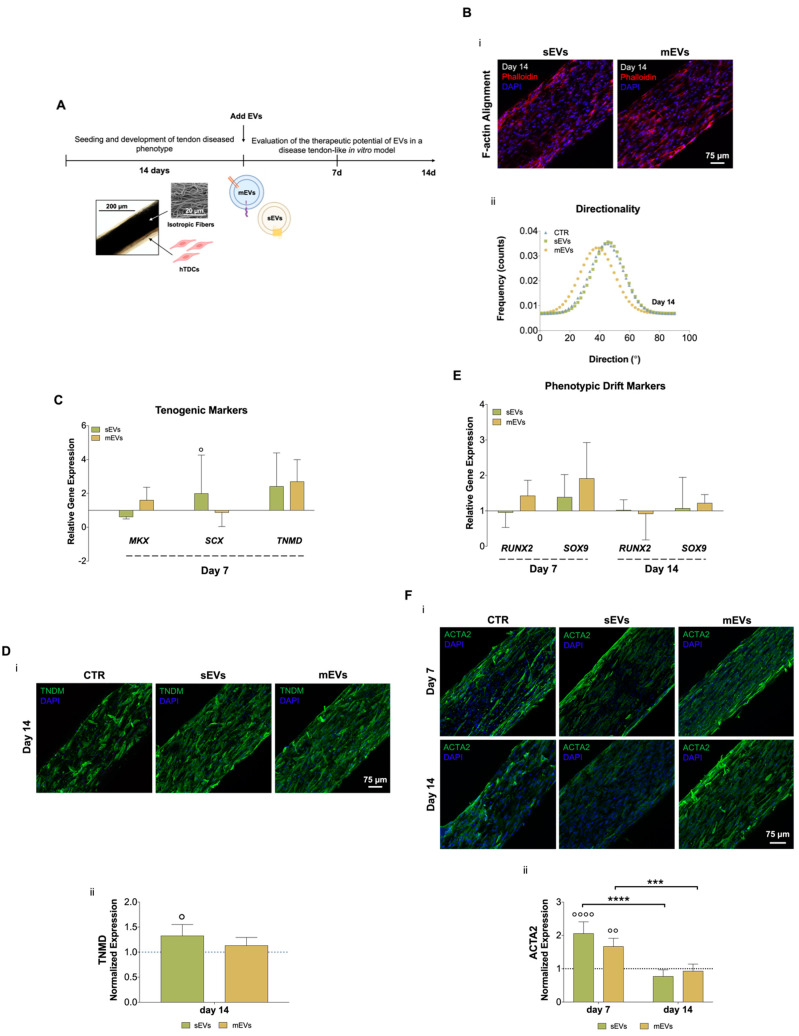
(**A**) Schematic representation of human tendon-derived stem cells (hTDCs) in culture over time. hTDCs were maintained in culture for 14 days to acquire a disease phenotype. Afterward, small extracellular vesicles (sEVs) and medium extracellular vesicles (mEVs) were added to the culture media of hTDCs to recover their healthy phenotype, which was evaluated after 7 and 14 days of culture. (**B**) (i) Confocal microscopy images of F-actin filaments (DAPI, blue; Phalloidin, red) of hTDCs supplemented with sEVs and mEVs after 14 days of culture. Scale bar: 75 µm. (ii) Representative directionality analysis of F-actin filaments in hTDCs after 14 days (n = 3). (**C**) Gene expression profile of tenogenic markers (mohawk (*MKX*), scleraxis (*SCX*), tenomodulin (*TNMD*)), after 7 days of culture (n = 4). Gene expression results are presented as fold changes with respect to the control group at each time point (represented by a line at 1). Statistical differences: ° *p* < 0.05 (against to the control). (**D**) (i) Confocal images of TNMD (green) at day 14. Images were counterstained with cells nuclei (DAPI, blue). Scale bar: 75 µm. (ii) TNMD fluorescence intensity quantitative analysis. Data were normalized to the control (1). Data are presented as mean ± standard deviation (SD) (n = 6). Statistical differences: ° *p* < 0.05 (against to the control). (**E**) Gene expression profile of phenotypic drift markers (runt-related transcription factor 2 (*RUNX2*) and SRY-Box transcription factor (*SOX9*)), after 7 and 14 days of culture. Gene expression results are presented as fold changes with respect to the control group at each time point (represented by a line at 1) (n = 4). (**F**) (i) Confocal micrographs of alpha-smooth muscle actin (ACTA2; green) at 7 and 14 days of culture. Nuclei were counterstained with DAPI (blue). Scale bar: 75 µm. (ii) Quantification of ACTA2 fluorescence intensity. Data were normalized to the control (1). Data are presented as mean ± SD (n = 6). Statistical differences: *** *p* < 0.001, **** *p* < 0.0001 (among days for the same group); °° *p* < 0.01, °°°° *p* < 0.0001 (against to the control).

In this work, building on our previous fibrous scaffolds for tendon tissue engineering and reconstruction [31,35,36], we developed a 3D tendon model that recreates its diseased state and used to test the potential of platelet EVs as a therapeutic option for the treatment of tendinopathy. For this, electrospun poly-ε-caprolactone (PCL) isotropic fiber threads were produced (Figure 1A). SEM images (Figure 1Ai) show that the fabricated threads have a diameter of 357.4 ± 16.12 µm, which is in the range of tendon fascicles [37]. Additionally, threads present a random nanotopography, and are composed of fibers with a diameter (1.85 ± 0.35 µm) within the primary collagen fiber range (Figure 1Aii) [32], mimicking the pathological tendon architecture and hierarchy. To include the cellular component in the 3D tendon model, composite living fibers (CLFs) were fabricated by coating electrospun threads with primary hTDCs encapsulated within a PL hydrogel shell. The hydrogel coating was used as a provisional ECM and, CLFs were maintained in culture for 14 days to allow the development of a diseased cell phenotype promoted by the synergy of PL biological signaling and contact with the random microtopography [38]. Along this time, the coating (69.82 ± 29.44 µm) retracted due to the fibrin matrix contraction (Figure 1Aiii, Supplementary Figure S1A), which promoted the contact of cells with the threads and the acquisition of a highly disorganized cellular orientation in response to the random microtopography (Supplementary Figure S1B). Moreover, in previous works, we showed that PL promoted a fast cellularization of the construct and the acquisition of a diseased cell phenotype while acting as a provisional ECM to allow the deposition of diseased-like ECM showing high collagen type I/type III ratio [38]. Therefore, we considered this time point as the initial state of the diseased tendon model used in subsequent sections to test the effects of the EVs (Figure 2A).

We isolated and quantified two different platelet EV populations. SEM micrographs show that both sEVs and mEVs have a spherical shape (Figure 1Bi). Nanoparticle tracking analysis (NTA) revealed size heterogeneity with an average hydrodynamic diameter of 132 ± 72 nm and 308 ± 147 nm for sEVs and mEVs, respectively. The number of EVs nanoparticles, also assessed using NTA, was of the same order of magnitude in both populations (2.32 × 10^9^ sEVs/mL and 1.16 × 10^9^ mEVs/mL, Figure 1Bii). To investigate the EV content in growth factors with potential roles in tendon homeostasis and hTDCs phenotype modulation, bFGF, TGF-β1, VEGF-A, and PDGF-BB were quantified (Figure 1Aiii). While mEVs were significantly enriched in TGF-β1, sEVs were enriched in bFGF, compared to the other population. Moreover, similar levels of PDGF-BB and VEGF-A were detected in both EV populations. These results are particularly interesting in light of the roles of these growth factors in tendon repair [32,39], which suggests a potential role of platelet EVs in the modulation of hTDCs phenotype.

### 2.2. Impact of Platelet EVs in the Diseased Tendon Cells Phenotype

To analyze the possible use of platelet-derived EVs as therapeutics for tendinopathies, both EV populations were added to the culture media of the diseased tendon in vitro model for 7 or 14 days (Figure 2A). We started by analyzing the effects of EVs on hTDCs morphology (namely, nuclei aspect ratio and F-actin alignment, which are related to cell shape and orientation, respectively) and proliferation. We did not observe any evident effect of both EV populations on cell morphology and proliferation after 7 and 14 days of culture (Supplementary Figure S1C). Additionally, after 14 days of culture with EVs, we could not see any evident effect on cells’ cytoskeleton alignment (Figure 2Bii; Supplementary Figure S1B). These results show that EVs had minor effects on cell morphology and organization in this short culture time frame.

We next evaluated the effect of platelet EVs on key tendon markers gene and protein expressions, such as scleraxis (SCX) and mohawk (MKX), two transcription factors expressed during tenocytes maturation, and tenomodulin (TNMD), a marker of mature tenocytes [40,41,42]. Gene expression analysis after 7 days of culture with EVs (Figure 2C) showed that sEVs significantly upregulated *SCX* expression compared with control. Moreover, both EV populations enhanced *TNMD* expression compared to the control group, although this difference was statistically non-significant. To confirm these results, immunostaining was used to assess TNMD protein expression after 14 days of culture (Figure 2Di). We observed TNMD expression increased in both EV stimulus groups compared with the control and, additionally, it was significantly higher in the sEVs than in the control group (Figure 2Dii). These results suggest that EV effects might promote the maintenance of healthy tendon cells phenotype. Furthermore, the expression profiles of genes related to osteoblasts and chondrocytes, which are associated with tenogenic drift/degeneration pathways [43,44], were also analyzed (Figure 2E). Levels of the osteogenic marker runt-related transcription factor 2 (*RUNX2*) and the chondrogenic marker SRY-Box transcription factor (*SOX9*) were downregulated along the time of culture with EVs, indicating that platelet EVs might contribute to lessen the tendon cells phenotypic drift typical of tendinopathy [43]. Furthermore, the levels of the myofibroblast marker alpha-smooth muscle actin (ACTA2), which is associated with the formation of scar/fibrotic tissue in tendon disorders [45], were quantified at the protein level (Figure 2Fi,ii). Although the expression of ACTA2 was significantly increased at early time points in the presence of platelet EVs, it showed a significant decrease over time, suggesting that platelet EVs favorably modulate tenocytes myofibroblastic phenotype activation and can contribute to promote a healthy tissue repair [45]. A possible explanation for these results might be related to the high content of growth factors in platelet EVs (Figure 1Biii). Although the TGF-β family has typically been related to ECM synthesis, it also plays central roles in early events of tendon development, tendon regeneration, and healthy tendon cell phenotype maintenance [46,47]. Moreover, bFGF has been shown to enhance tenomodulin expression in tenocyte precursors [48]. Additionally, EVs, including those derived from platelets [49], which have been shown to contain microRNAs that are responsible for important roles in inflammation, fibrogenesis, and tissue repair, and its administration has produced positive effects on tendon healing [50]. Thus, future studies should focus on the characterization of microRNAs profiles in our platelet EVs to further analyze their possible contribution in tendon healing mechanisms. In summary, these results indicate that both platelet EV populations might promote the recovery or maintenance of the tenogenic phenotype by reducing the typical phenotypic drift of tendon cells towards fibrotic or osteogenic lineages commonly observed in tendinopathy.

### 2.3. Platelet EVs Modulate ECM Deposition and Remodeling by hTDCs

**Figure 3:**
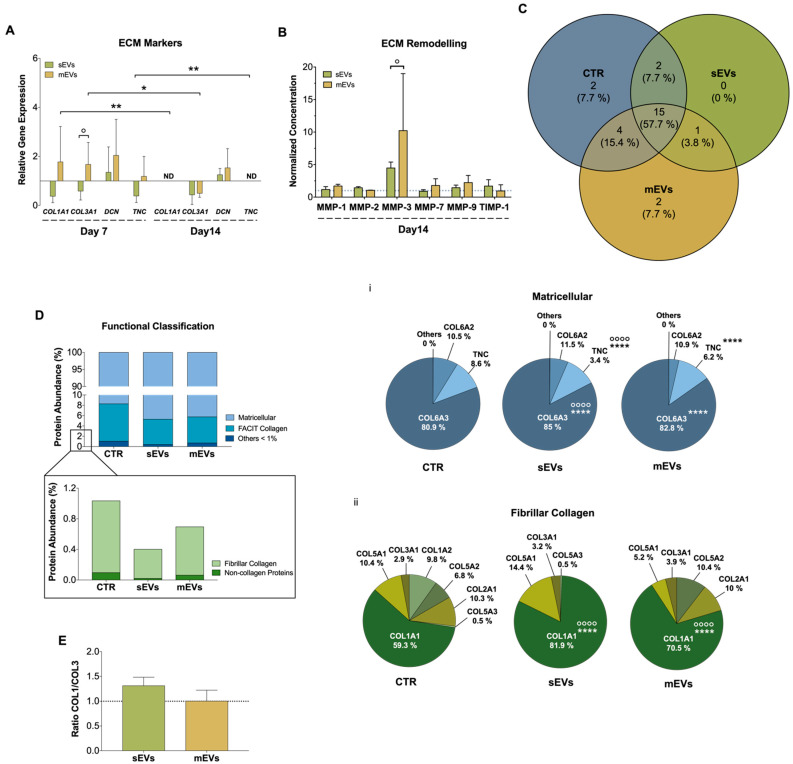
(**A**) Gene expression of tendon-related extracellular matrix (ECM) markers, collagen type I alpha 1 chain (*COL1A1*), collagen type III alpha 1 chain (*COL3A1*), decorin (*DCN*), and tenascin (*TNC*), after 7 and 14 days with small extracellular vesicles (sEVs) and medium extracellular vesicles (mEVs). Expression of target genes normalized against the reference genes glyceraldehyde-3-phosphate dehydrogenase (*GADPH*) and glucuronidase beta (*GUSB*) and gene expression normalized at the control of each day (1) (n = 4). Statistical differences: * *p* < 0.05, ** *p* < 0.01 (among days for the same group); ° *p* < 0.05 (sEVs against mEVs). (**B**) Concentration of matrix metalloproteinase-1, -2, -3, -7 and -9 (MMP-1, MMP-2, MMP-3, MMP-7, and MMP-9) and tissue inhibitor of metalloproteinase-1 (TIMP-1), present in the 14 days culture medium. Data were normalized to the control of day 14 (unstimulated cells, set to 1) (n = 4). Statistical differences: ° *p* < 0.05 (sEVs against mEVs). (**C**) Venn diagram of common and unique ECM proteins identified in the isotropic fiber-encapsulated human tendon-derived stem cell (hTDC) system, non-supplemented and supplemented with sEVs and mEVs at 14 days of culture (n = 3). (**D**) Relative abundance of ECM proteins was categorized according to their function. Quantitative assessment of the ECM proteomic profile revealed (i) matricellular and (ii) fibrillar collagen are the most abundant categories (n = 3). Statistical differences: °°°° *p* < 0.0001 (against control), **** *p* < 0.0001 (sEVs against mEVs) (**E**) Proteomic profile assessment of collagen type I and collagen type III (COL1/COL3) ratio at 14 days of culture (n = 3). Results were normalized to control that was set to 1.

Tendon is a connective tissue composed predominantly of collagen type I, and other lower proportions of collagen subtypes such as collagen type III, V, and XII [51]. Moreover, there are other constituents of tendon ECM like decorin (DCN), an important proteoglycan during collagen fibrillogenesis, and tenascin-C (TCN), a glycoprotein responsible for growth factor activity modulation and cells-ECM interaction [52,53]. Dysregulated ECM homeostasis is one of the main features of tendon disorders involving loss of collagen organization and unbalanced ECM turnover [1,54]. During this process, a higher production of type III collagen takes place to protect the injured area, which is accompanied by an increased production of metalloproteinases (MMPs) and tissue inhibitors of MMPs (TIMPs) to modulate the ECM remodeling [1]. To evaluate the effect that platelet EVs have in hTDCs ECM production and remodeling, we assessed the gene expression of different ECM markers (Figure 3A). In general, mEVs induced an initial (7 days) upregulation in the expression of type I and III collagens (*COL1A1*, *COL3A1*), *DCN* and *TNC*, while sEVs showed an opposite effect. These results might also be related with an upregulation by TGF-β, a potent stimulator of ECM synthesis [55], which was higher in mEVs than in sEVs (Figure 1Biii). Nonetheless, at later culture times (14 days), both platelet EVs significantly downregulated the gene expression of these ECM markers, except for DCN. Furthermore, we also analyzed the effects of platelet EVs on the cell secretion levels of different MMPs and TIMP1 (Figure 3B). We found that after 14 days of culture, MMP-3, responsible for degrading minor tendon ECM components such as collagen type III and proteoglycans [56], showed increased levels, albeit statistically non-significant, in the presence of both platelet EVs in comparison with the control. The decreased COL3A1 expression together with the increased detected levels of MMP-3 may suggest that the initial more fibrotic-like ECM, characterized by higher collagen type III deposition [57,58,59], is being remodeled into a healthier ECM.

To support this hypothesis and to have a more comprehensive understanding of the impact of platelet EVs in ECM production and remodeling, we next performed a proteomic analysis. For this, the model constructs were treated with EVs for 14 days before being decellularized in order to remove their cellular content and better recover and identify their respective ECM components [60]. A total of 26 ECM proteins were identified (*p* ≤ 0.05; two unique peptides, in at least three replicates) (Table S1), in which 15 (~58%) ECM proteins were common to all groups (Figure 3C, Table S2). Additionally, to better categorize the role of these proteins, a Gene Ontology (GO) analysis was performed. We observed that matricellular proteins and fibril-associated collagens with interrupted triple helices (FACIT) are the major categories identified, constituting more than 90% and 8% of the proteins, respectively, while other minor proteins such as fibrillar collagens, basement membrane proteins and structural and regulator ECM proteins, were also identified (Figure 3D). Matricellular proteins are a group of non-structural proteins present in the ECM with key roles in the modulation of cell function and cell adhesion [61]. Among these, COL6A3, which provides a link among the structural constituents of the ECM and cells [62], was the major matricellular protein identified in all the groups (Figure 3Di). The abundance of this collagen was significantly higher in sEVs than in the mEVs and control groups (Figure 3Di). Moreover, the levels of TNC were significantly lower in the groups supplemented with platelet sEVs than in the mEVs and control groups, in line with gene expression results. Since TNC is upregulated upon tissue injury, inflammation and tissue remodeling [63], its decrease might be related with a healthier tendon ECM. Regarding the fibrillar collagens, which constitute the structural elements of ECM, COL1A1 showed the highest proportion in all groups (Figure 3Dii). Remarkably, the abundance of COL1A1 was significantly greater in the sEVs and mEVs groups than in the control group, and in sEVs compared with mEVs. In addition, the levels of COL3A1 were similar in all groups. This generates a (non-significant) increased ratio of type I/type III collagen (all chains accounted) in groups treated with platelet sEVs compared with the control (Figure 3E), which has been related to a healthier tendon ECM [61]. Intriguingly, although mEVs promoted the expression of ECM markers compared with sEVs (Figure 3A), the continuous activation with TGF-β, which has been related to tissue fibrosis, produced a more fibrotic-like response. This is also in agreement with the higher content of bFGF in sEVs than in mEVs, which has been shown to inhibit the activation of pro-fibrotic myofibroblasts [64]. Overall, these results suggest that platelet sEVs have the potential to promote the remodeling and synthesis of a healthy tendon ECM.

### 2.4. Modulation of Inflammatory Markers in hTDCs by Platelet EVs

**Figure 4:**
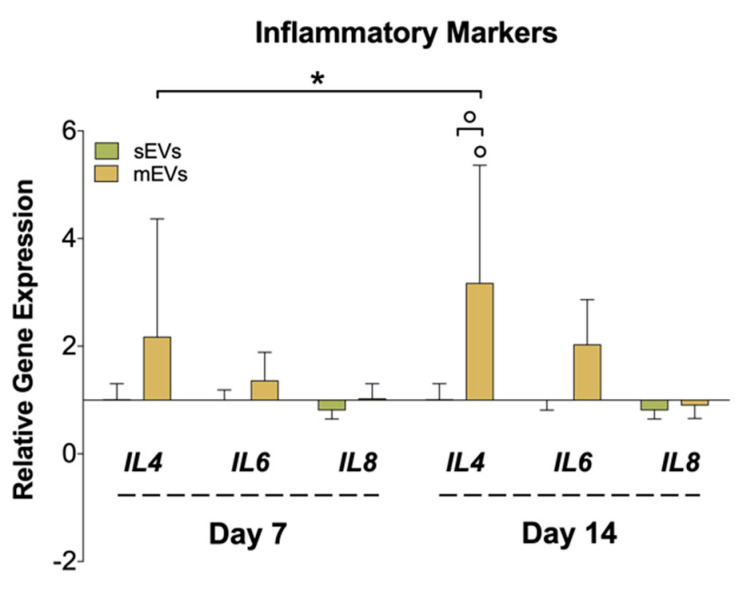
Gene expression of inflammatory markers (interleukin-4; -6; -8 (*IL4*, *IL6,* and *IL8*)), after 7 and 14 days of culture with EVs. The target gene expression was normalized against glyceraldehyde-3-phosphate dehydrogenase (*GAPDH*) and glucuronidase beta (*GUSB)* and the data presented for the non-supplemented system each day (1) (n = 4). Statistical significances: * *p* < 0.05 (among days for the same group); ° *p* < 0.05 (between sEVs and mEVs or against the control for the same day).

Chronic inflammation is another hallmark of tendon disorders, and is highly related to the inflammatory mediators released to the tendinopathy microenvironment by tenocytes, among other cells [65]. To assess the effect of platelet EVs in modulating the inflammatory response in hTDCs, we analyzed the gene expression of different cytokines (Figure 4). We observed a significant upregulation in the expression of the anti-inflammatory cytokine interleukin 4 (*IL4*) in samples treated with mEVs compared with sEVs and control groups. Recent works have proved that tenocytes participate in the amplification of the inflammatory response after tendon damage through the secretion of different cytokines and chemokines [66]. On the other hand, IL-4 promotes the transformation of naive CD4 T cells and macrophages into a Th2 T cell or M2 macrophage phenotype, respectively, which drives the type 2 anti-inflammatory immune response [67]. Thus, it is plausible that tenocytes can also have a role in the modulation of the reparative (anti-inflammatory) immune response, which should be studied in future works. Interestingly, IL-4 has been suggested as a potential tendon-healing therapeutic agent [68]. On the other hand, we could only observe minor differences in the expression of the pro-inflammatory *IL6* and *IL8* upon treatment with platelet EVs. Taken together with these outcomes, platelet mEVs might also have an influence in the mediation/regulation of inflammatory pathways potentiating the inflammation resolution.

## 3. Materials and Methods

### 3.1. Production and Characterization of Isotropic Fiber Threads

Poly-ε-caprolactone (PCL, average MW 80,000, Sigma-Aldrich, Saint Louis, MO, USA) electrospinning solution was prepared with 17% (*w*/*v*) of PCL dissolved in a chloroform (Honeywell, Charlotte, NC, USA)/N, N’-dimethylformamide (DMF, Carlo Erba Reagents, Val de Reuil, France) (*v*/*v*, 7:3) solution, under agitation overnight at room temperature (RT). To produce the PCL misaligned fiber threads, a customized electrospinning device set up was used [36]. Succinctly, a syringe with a 21 G needle was filled with the PCL solution and jetted, under a constant flow rate of 1.0 mL/h and voltage of 8.0–9.0 kV, into a 20% (*v*/*v*) ethanol/water bath. The needle was placed 13 cm above the surface of the bath, and a roller with a constant speed of 0.14 cm/s was used to collect the threads. During the fibers production procedure, the temperature was maintained at 21–23 °C with a relative humidity of 43–45%.

The morphology of the threads was assessed by high-resolution scanning electron microscopy (SEM, JSM-6010 LV, JEOL, Tokyo, Japan) operating at an accelerating voltage of 10 kV. Briefly, samples were placed onto an adhesive carbon film and coated with gold under vacuum for one minute (Cressington, Watford, UK) before image acquisition. Diameters of threads and fibers (n = 50), and their alignment (directionality analysis) were determined using ImageJ 1.520 (NIH, Bethesda, MD, USA) software. Fiber’s 3D holders were produced using a 3D printer (see Supplementary Material).

### 3.2. Isolation and Characterization of Platelet-Derived EVs

A pool of 100 healthy donors of platelet concentrates (PC) was provided by Serviço de Imunohemoterapia do Centro Hospitalar de São João (CHUSJ, Porto, Portugal) under an ethical commission approval (No. 363/18). To obtain platelet EVs, the PC pool was first submitted to three freeze/thaw cycles in liquid nitrogen followed by thawing at 37 °C. In the last cycle, the PC pool was subjected to two initial centrifugations at 2000× *g* for 30 min at 4 °C and 12,000× *g* for 45 min at 4 °C (FA-45-6-30 rotor, Eppendorf 5810 R, Eppendorf, Hamburg, Germany) to discard cell debris and to recover the medium EVs (mEVs), respectively. Afterward, the supernatant of the last centrifugation cycle was ultracentrifuged at 110,000× *g* for 2 h at 4 °C (S52-ST rotor, ThermoFisher Scientific, Sorvall Mx 120 Plus, Micro-ultracentrifuge, Waltham, MA, USA) to pellet small EVs (sEVs). The sEV pellets were washed in phosphate-buffered saline (PBS; Sigma-Aldrich, Saint Louis, MO, USA) and then ultracentrifuged at 110,000× *g* for 70 min at 4 °C [69]. The resulting EV pellets were re-suspended in 1 mL of ultrapure water and stored at −80 °C until further use.

EVs morphology was assessed by high-resolution SEM (Auriga CompactLV, Zeiss, Oberkochen, Germany). Briefly, EVs were fixed with 2.5% glutaraldehyde in PBS and dehydrated in an ascending series of ethanol at 50%, 70%, 90%, and 100% onto a glass substrate. Size distribution of EVs and particle concentration were measured using NTA instrument (NS500, Nanosight, Malvern, Worcestershire, UK) equipped with an EMCCD camera and 488 nm laser. The concentration of growth factors, bFGF, TGFβ-1, VEGF-A, and PDGF-BB, within EVs was quantified by enzyme-linked immunosorbent assay (ELISA) using the commercial DuoSet ELISA kits (R&D Systems, Minneapolis, MN, USA), following suppliers’ specifications (n = 3).

### 3.3. Human Tendon-Derived Cell Isolation and Encapsulation

Human tendon-derived cells were isolated from Sartorius tendon tissue samples from healthy male patients with ages between 25–30 years undergoing elective orthopedic surgeries at the Hospital da Prelada (Porto, Portugal) under protocols implemented and approved by the Hospital Ethical Committee (No. 005/2019). To perform hTDC isolation, a protocol described elsewhere was followed [30,70]. Cells were used at passages 3–4.

3D-printed molds with four misaligned fiber replicates placed in 6-well plates were sterilized in 70% (*v*/*v*) ethanol for 30 min followed by 30 min of ultraviolet radiation. Afterward, isotropic threads were immersed in a solution of 10 U/mL thrombin from bovine plasma (40–30 NIH units/mg protein, Sigma-Aldrich, Saint Louis, MO, USA) in 5 mM calcium chloride (CaCl_2_, MERCK KGaA, Darmstadt, Germany) for 45 min at RT [38,71]. Then, threads were transferred to a new channel and coated with a solution of hTDCs in PL hydrogel, produced as described previously [71,72], at a density of 2.5 × 10^5^ cells/mL for 2 h at 37 °C in a 5% humidity atmosphere for PL gelation. PL, before use, was previously thawed at 37 °C and centrifuged at 3000× *g* for 10 min at 4 °C (FA-45-6-30 rotor, Eppendorf 5810 R, Eppendorf, Hamburg, Germany) for cell debris removal. After 2 h incubation, channel supports were removed and a complete culture medium consisting of α-MEM (ThermoFisher Scientific, Waltham, MA, USA) supplemented with 10% (*v*/*v*) of fetal bovine serum (FBS, Life Technologies) and 1% (*v*/*v*) of antibiotic/antimycotic (A/A) was added and the system was maintained in culture for 14 days to allow hTDCs to acquire a diseased phenotype. PL hydrogel thickness was observed by optical microscopy (CKX53, Olympus, Tokyo, Japan) and analyzed by ImageJ 1.520 (NIH, Bethesda, MD, USA) software.

After 14 days of culture, the cell-coated threads (diseased-like tendon model) were incubated with 100 µg/mL of sEVs and mEVs in an EV-depleted culture medium consisting of α-MEM (ThermoFisher Scientific, Waltham, MA, USA) supplemented with 10% (*v*/*v*) of EV-depleted FBS (ThermoFisher Scientific, Waltham, MA, USA) and 1% (*v*/*v*) of A/A. Threads cultured without EV supplementation were used as controls.

### 3.4. hTDC Morphology and Actin Filament Organization

Samples were fixed with 10% (*v*/*v*) formalin (Bio-Optica, Milan, Italy) for 30 min at RT. Cell cytoskeleton and nuclei were stained with phalloidin-tetramethylrhodamine B isothiocyanate (Sigma-Aldrich, Saint Louis, MO, USA) and diamidino-2-phenylindole (DAPI; VWR, Radnor, PA, USA) for 45 min at RT, respectively. Samples were analyzed through confocal laser scanning microscopy (CLSM, TCS SP8, Leica, Wetzlar, Germany) and images were processed using LAS X software from Leica. 

Actin filament alignment was assessed through directionality analysis (n = 3). Moreover, a total of 400–450 nuclei were measured using different confocal images of each condition (n = 3) to determine the nuclei aspect ratio by dividing its length by the width. Lastly, the number of nuclei *per* area of hTDCs was calculated to assess cell proliferation. All these analyses were performed using ImageJ 1.520 (NIH, Bethesda, MD, USA) software.

### 3.5. Immunocytochemistry of Encapsulated hTDCs

Samples (n = 5–6) were fixed with 10 (*v*/*v*)% formalin and permeabilized with 0.1 (*v*/*v*)% X-100 Triton (ThermoFisher Scientific, Waltham, MA, USA)/PBS for 10 min at RT. After PBS rinsing, non-specific binding was blocked with 2.5% normal horse serum (Vector Laboratories, Burlingame, CA, USA) for 1 h at RT. Then, samples were incubated overnight at 4 °C with primary antibodies diluted in 0.1% BSA/PBS against ACTA2 (rabbit, ab32575, Abcam, Cambridge, UK), and anti-TNMD (rabbit polyclonal) generated against TNMD C-terminus (237-317 aa) provided by Prof. Denitsa Docheva (produced in co-operation with Metabion International, Planegg, Germany, PAB 201603-00002). Subsequently, samples were washed in PBS, incubated with 0.3% (*v*/*v*) hydrogen peroxide (PanReac AppliChem, Barcelona, Spain) for 15 min at RT, and incubated with anti-rabbit AlexaFluor 488 fluorescent secondary antibody (ThermoFisher Scientific, Waltham, MA, USA) for 1 h at RT. Finally, nuclei and F-actin filaments were counterstained with DAPI and phalloidin for 30 min at RT, respectively. Immunolabeled samples were analyzed through confocal laser scanning microscopy CLSM, TCS SP8, Leica, Wetzlar, Germany) and the quantitative analysis of the immunofluorescence images was performed using ImageJ 1.520 (NIH, Bethesda, MD, USA) software.

### 3.6. mRNA Extraction and Reverse Transcriptase Quantitative Polymerase Chain Reaction (RT-qPCR)

Total mRNA (n = 4) was isolated with RNeasy Mini Kit (Qiagen, Hilden, Germany) according to the manufacturer’s protocol and quantified using Nanodrop spectrophotometer (ND-1000, ThermoFisher Scientific, Waltham, MA, USA). Complementary DNA (cDNA) was synthesized through qScript cDNA Synthesis Kit (Quanta Biosciences, Beverly, MA, USA) following the supplier’s instructions. Furthermore, RT-PCR was carried out to quantify the expression of the genes presented in Table S3 using as reference genes glyceraldehyde-3-phosphate dehydrogenase (*GAPDH*) and glucuronidase beta (*GUSB*) through PerfeCTA SYBR Green FastMix kit (Quanta Biosciences, Beverly, MA, USA) according to protocol kit instructions. Blanks were considered as negative controls for each primer. Relative expression was normalized initially against reference genes followed by the average of hTDCs control samples at each time point, calculated according to the ∆∆Ct method [73].

### 3.7. ECM Protein Proteomic Analysis

After 14 days of culture with EVs, samples were first decellularized to extract the ECM proteins (n = 3). Briefly, samples were washed in 0.5% (*v*/*v*) X-100 Triton in PBS for 2 h at 37 °C followed by PBS overnight incubation at 4 °C. Finally, the threads were resuspended in a solution of 4% of sodium dodecyl sulphate (SDS, Sigma-Aldrich, USA), 1:100 protease inhibitor (Sigma-Aldrich, Saint Louis, MO, USA), 0.1 M dithiothreitol (DTT, abcr Company, Karlsruhe, Germany) in 0.1 M Tris-HCl and vigorously vortexed to detach ECM proteins from the threads. Afterward, each protein sample was processed for proteomics analysis following the procedure described in [74] with the solid-enhanced sample preparation (SP3) [75]. Enzymatic digestion was performed with Trypsin/LysC (2 μg) overnight at 37 °C at 1000 rpm using an Thermomixer Comfort (Eppendorf, Hamburg, Germany). Protein identification and quantitation was performed by label-free quantification. The raw data was processed using Proteome Discoverer 2.5 software (Thermo Scientific, Waltham, MA, USA) and searched against the UniProt database for the *Homo sapiens* Proteome 2019_09 (74.034 sequences).

Data analysis was performed only for ECM proteins expressing a ρ-value ≤ 0.05, and at least 2 unique peptides were analyzed. ECM proteins were categorized considering their biological processes and cellular components through Gene Ontology (GO). Protein Analysis Through Evolutionary Relationships (PANTHER; version 15.0) was used to perform GO enrichment analysis.

### 3.8. Multiplex Immunoassay 

A custom-made Human Procarta Plex 5-Plex (ThermoFisher Scientific, Austria) was used to quantify (MMP)-1, 2, 3, 8, 9, and 13 and a Human ProcartaPlex Simplex Kit to quantify TIMP-1 (ThermoFisher Scientific, Waltham, MA, USA) released to culture media after 14 days of culture (n = 4), according to manufacturer’s instructions. The concentrations of each analyte were determined using MAGPIX Instrument System (Luminex, Austin, TX, USA) and calculated by Luminex xPONENT 4.2 software.

### 3.9. Statistical Analysis

Statistical analysis was performed using GraphPad PRISM 7.0 software (San Diego, CA, USA). Results are presented as values ± SD (standard deviation) unless otherwise stated. One-way or two-way analysis of variance (ANOVA) was performed in parametric distributions by the Bonferroni post hoc test for multiple comparisons, on the other hand, the Mann–Whitney test was used for non-parametric tests. Statistical significance was set to *p*-value < 0.05 with a confidence interval of 95%.

## 4. Conclusions

This work sought to study the therapeutic potential of two populations of platelet EVs in a 3D in vitro tendon disease model. We verified that although EVs do not have a remarkable influence on hTDC morphology, they are able to influence their biological response. We showed that the interaction of platelet sEVs with diseased-like hTDCs promotes the recovery of their tenogenic phenotype, increasing the expression of tendon-related markers and decreasing the expression of osteogenic and fibrotic markers. Moreover, treatment with platelet sEVs promoted the remodeling and deposition of a healthier ECM in the constructs, showing increased collagen type I/type III ratio. Finally, we also showed that platelet mEVs increased the expression of anti-inflammatory mediators, which might contribute to blunting the inflammatory processes occurring in the injured or tendinopathic tissue. Overall, the results showed that while platelet sEVs have a positive influence on the recovery of hTDCs healthy phenotype by increasing the expression of tendon cells markers, and promoting ECM remodeling, platelet mEVs increase the expression of anti-inflammatory cytokines, suggesting that platelet EVs might be a promising therapeutic tool for tendon injury recovery. The beneficial effects of these vesicles are worthy to be explored in further studies to provide more insights on how EVs interact with cells and to further understand their effect on tendon disorders.

## Data Availability

Not applicable.

## Supplementary Materials

The following supporting information can be downloaded at: https://www.mdpi.com/article/10.3390/ijms23062948/s1.

## References

[1] Millar N.L., Silbernagel K.G., Thorborg K., Kirwan P.D., Galatz L.M., Abrams G.D., Murrell G.A.C., McInnes I.B., Rodeo S.A. (2021). Tendinopathy. Nat. Rev. Dis. Prim..

[2] Millar N.L., Murrell G.A.C., McInnes I.B. (2017). Inflammatory mechanisms in tendinopathy—Towards translation. Nat. Rev. Rheumatol..

[3] Leadbetter W.B. (2005). Anti-inflammatory therapy in tendinopathy: The role of nonsteroidal drugs and corticosteroid injections. Tendon Inj. Basic Sci. Clin. Med..

[4] Aicale R., Bisaccia R.D., Oliviero A., Oliva F., Maffulli N. (2020). Current pharmacological approaches to the treatment of tendinopathy. Expert Opin. Pharmacother..

[5] Mishra A., Woodall J., Vieira A. (2009). Treatment of Tendon and Muscle Using Platelet-Rich Plasma. Clin. Sports Med..

[6] Zhou Y., Wang J.H.C. (2016). PRP Treatment Efficacy for Tendinopathy: A Review of Basic Science Studies. Biomed Res. Int..

[7] Lomas A.J., Ryan C.N.M., Sorushanova A., Shologu N., Sideri A.I., Tsioli V., Fthenakis G.C., Tzora A., Skoufos I., Quinlan L.R. (2015). The past, present and future in scaffold-based tendon treatments. Adv. Drug Deliv. Rev..

[8] Padilla S., Sánchez M., Orive G., Anitua E. (2017). Human-based biological and biomimetic autologous therapies for musculoskeletal tissue regeneration. Trends Biotechnol..

[9] Yáñez-Mó M., Siljander P.R.-M., Andreu Z., Zavec A.B., Borràs F.E., Buzas E.I., Buzas K., Casal E., Cappello F., Carvalho J. (2015). Biological properties of extracellular vesicles and their physiological functions. J. Extracell. Vesicles.

[10] Silva A.M., Teixeira J.H., Almeida M.I., Gonçalves R.M., Barbosa M.A., Santos S.G. (2017). Extracellular Vesicles: Immunomodulatory messengers in the context of tissue repair/regeneration. Eur. J. Pharm. Sci..

[11] Riazifar M., Pone E.J., Lötvall J., Zhao W. (2017). Stem cell extracellular vesicles: Extended messages of regeneration. Annu. Rev. Pharmacol. Toxicol..

[12] Van Niel G., D’Angelo G., Raposo G. (2018). Shedding light on the cell biology of extracellular vesicles. Nat. Rev. Mol. Cell Biol..

[13] Théry C., Ostrowski M., Segura E. (2009). Membrane vesicles as conveyors of immune responses. Nat. Rev. Immunol..

[14] Wiklander O.P.B., Brennan M.Á., Lötvall J., Breakefield X.O., EL Andaloussi S. (2019). Advances in therapeutic applications of extracellular vesicles. Sci. Transl. Med..

[15] García-Manrique P., Matos M., Gutiérrez G., Pazos C., Blanco-López M.C. (2018). Therapeutic biomaterials based on extracellular vesicles: Classification of bio-engineering and mimetic preparation routes. J. Extracell. Vesicles.

[16] Agrahari V., Agrahari V., Burnouf P.A., Chew C.H., Burnouf T. (2019). Extracellular Microvesicles as New Industrial Therapeutic Frontiers. Trends Biotechnol..

[17] Mendes B.B., Gómez-Florit M., Babo P.S., Domingues R.M., Reis R.L., Gomes M.E. (2018). Blood derivatives awaken in regenerative medicine strategies to modulate wound healing. Adv. Drug Deliv. Rev..

[18] Amable P., Carias R.B., Teixeira M.V., da Cruz Pacheco Í., Corrêa do Amaral R.J., Granjeiro J., Borojevic R. (2013). Platelet-rich plasma preparation for regenerative medicine: Optimization and quantification of cytokines and growth factors. Stem Cell Res. Ther..

[19] De Vos R.J., Windt J., Weir A. (2014). Strong evidence against platelet-rich plasma injections for chronic lateral epicondylar tendinopathy: A systematic review. Br. J. Sports Med..

[20] Scott A., LaPrade R.F., Harmon K.G., Filardo G., Kon E., Della Villa S., Bahr R., Moksnes H., Torgalsen T., Lee J. (2019). Platelet-Rich Plasma for Patellar Tendinopathy: A Randomized Controlled Trial of Leukocyte-Rich PRP or Leukocyte-Poor PRP Versus Saline. Am. J. Sports Med..

[21] Liddle A.D., Rodríguez-Merchán E.C. (2015). Platelet-Rich Plasma in the Treatment of Patellar Tendinopathy: A Systematic Review. Am. J. Sports Med..

[22] Johnson J., Wu Y.W., Blyth C., Lichtfuss G., Goubran H., Burnouf T. (2021). Prospective Therapeutic Applications of Platelet Extracellular Vesicles. Trends Biotechnol..

[23] Torreggiani E., Perut F., Roncuzzi L., Zini N., Baglìo S., Baldini N. (2014). Exosomes: Novel effectors of human platelet lysate activity. Eur. Cells Mater..

[24] Cancedda R., Bollini S., Descalzi F., Mastrogiacomo M., Tasso R. (2017). Learning from Mother Nature: Innovative Tools to Boost Endogenous Repair of Critical or Difficult-to-Heal Large Tissue Defects. Front. Bioeng. Biotechnol..

[25] Antich-Rosselló M., Forteza-Genestra M.A., Ramis J.M., Monjo M. (2021). Platelet-Derived Extracellular Vesicles for Regenerative Medicine. Int. J. Mol. Sci..

[26] Crespo-Diaz R., Behfar A., Butler G.W., Padley D.J., Sarr M.G., Bartunek J., Dietz A.B., Terzic A. (2011). Platelet Lysate Consisting of a Natural Repair Proteome Supports Human Mesenchymal Stem Cell Proliferation and Chromosomal Stability. Cell Transplant..

[27] Lee N.M., Erisken C., Iskratsch T., Sheetz M., Levine W.N., Lu H.H. (2017). Polymer fiber-based models of connective tissue repair and healing. Biomaterials.

[28] Schoenenberger A.D., Foolen J., Moor P., Silvan U., Snedeker J.G. (2018). Substrate fiber alignment mediates tendon cell response to inflammatory signaling. Acta Biomater..

[29] Schoenenberger A.D., Tempfer H., Lehner C., Egloff J., Mauracher M., Bird A., Widmer J., Maniura-Weber K., Fucentese S.F., Traweger A. (2020). Macromechanics and polycaprolactone fiber organization drive macrophage polarization and regulate inflammatory activation of tendon in vitro and in vivo. Biomaterials.

[30] Domingues R.M.A., Chiera S., Gershovich P., Motta A., Reis R.L., Gomes M.E. (2016). Enhancing the Biomechanical Performance of Anisotropic Nanofibrous Scaffolds in Tendon Tissue Engineering: Reinforcement with Cellulose Nanocrystals. Adv. Healthc. Mater..

[31] Tomás A.R., Goncąlves A.I., Paz E., Freitas P., Domingues R.M.A., Gomes M.E. (2019). Magneto-mechanical actuation of magnetic responsive fibrous scaffolds boosts tenogenesis of human adipose stem cells. Nanoscale.

[32] Docheva D., Müller S.A., Majewski M., Evans C.H. (2015). Biologics of Tendon Repair. Adv. Drug Deliv. Rev..

[33] Steinmann S., Pfeifer C.G., Brochhausen C., Docheva D. (2020). Spectrum of tendon pathologies: Triggers, trails and end-state. Int. J. Mol. Sci..

[34] Rolf C.G., Fu B.S.C., Pau A., Wang W., Chan B. (2001). Increased cell proliferation and associated expression of PDGFRβ causing hypercellularity in patellar tendinosis. Rheumatology.

[35] Almeida H., Domingues R.M.A., Mithieux S.M., Pires R.A., Gonçalves A.I., Gómez-Florit M., Reis R.L., Weiss A.S., Gomes M.E. (2019). Tropoelastin-Coated Tendon Biomimetic Scaffolds Promote Stem Cell Tenogenic Commitment and Deposition of Elastin-Rich Matrix. ACS Appl. Mater. Interfaces.

[36] Laranjeira M., Domingues R.M.A., Costa-Almeida R., Reis R.L., Gomes M.E. (2017). 3D Mimicry of Native-Tissue-Fiber Architecture Guides Tendon-Derived Cells and Adipose Stem Cells into Artificial Tendon Constructs. Small.

[37] Kannus P. (2000). Structure of the tendon connective tissue. Scand. J. Med. Sci. Sport..

[38] Calejo I., Labrador-Rached C.J., Gomez-Florit M., Docheva D., Reis R.L., Domingues R.M.A., Gomes M.E. Bioengineered 3D living fibers as in vitro human tissue models of tendon physiology and pathology.

[39] Anitua E., Alkhraisat M.H., Orive G. (2012). Perspectives and challenges in regenerative medicine using plasma rich in growth factors. J. Control. Release.

[40] Shukunami C., Takimoto A., Nishizaki Y., Yoshimoto Y., Tanaka S., Miura S., Watanabe H., Sakuma T., Yamamoto T., Kondoh G. (2018). Scleraxis is a transcriptional activator that regulates the expression of Tenomodulin, a marker of mature tenocytes and ligamentocytes. Sci. Rep..

[41] Docheva D., Hunziker E.B., Fassler R., Brandau O. (2005). Tenomodulin Is Necessary for Tenocyte Proliferation and Tendon Maturation. Mol. Cell. Biol..

[42] Ito Y., Toriuchi N., Yoshitaka T., Ueno-Kudoh H., Sato T., Yokoyama S., Nishida K., Akimoto T., Takahashi M., Miyaki S. (2010). The Mohawk homeobox gene is a critical regulator of tendon differentiation. Proc. Natl. Acad. Sci. USA.

[43] Zhang J., Wang J.H.C. (2010). Mechanobiological response of tendon stem cells: Implications of tendon homeostasis and pathogenesis of tendinopathy. J. Orthop. Res..

[44] Zhang C., Zhu J., Zhou Y., Thampatty B.P., Wang J.H.C. (2019). Tendon Stem/Progenitor Cells and Their Interactions with Extracellular Matrix and Mechanical Loading. Stem Cells Int..

[45] Zhao W., Wang X., Sun K.H., Zhou L. (2018). α-Smooth Muscle Actin Is Not a Marker of Fibrogenic Cell Activity in Skeletal Muscle Fibrosis. PLoS ONE.

[46] Kaji D.A., Howell K.L., Balic Z., Hubmacher D., Huang A.H. (2020). TGFβ signaling is required for tenocyte recruitment and functional neonatal tendon regeneration. eLife.

[47] Tan G.K., Pryce B.A., Stabio A., Brigande J.V., Wang C., Xia Z., Tufa S.F., Keene D.R., Schweitzer R. (2020). TGFβ signaling is critical for maintenance of the tendon cell fate. eLife.

[48] Tokunaga T., Shukunami C., Okamoto N., Taniwaki T., Oka K., Sakamoto H., Ide J., Mizuta H., Hiraki Y. (2015). FGF-2 Stimulates the Growth of Tenogenic Progenitor Cells to Facilitate the Generation of Tenomodulin—Positive Tenocytes in a Rat Rotator Cuff Healing Model. Am. J. Sports Med..

[49] Tan M., Yan H.-B., Li J.-N., Li W.-K., Fu Y.-Y., Chen W., Zhou Z. (2016). Thrombin Stimulated Platelet-Derived Exosomes Inhibit Platelet-Derived Growth Factor Receptor-Beta Expression in Vascular Smooth Muscle. Cell. Physiol. Biochem..

[50] Dubin J.A., Greenberg D.R., Iglinski-Benjamin K.C., Abrams G.D. (2018). Effect of micro-RNA on tenocytes and tendon-related gene expression: A systematic review. J. Orthop. Res..

[51] Banos C.C., Thomas A.H., Kuo C.K. (2008). Collagen fibrillogenesis in tendon development: Current models and regulation of fibril assembly. Birth Defects Res. Part C-Embryo Today Rev..

[52] Zhang G., Ezura Y., Chervoneva I., Robinson P.S., Beason D.P., Carine E.T., Soslowsky L.J., Iozzo R.V., Birk D.E. (2006). Decorin regulates assembly of collagen fibrils and acquisition of biomechanical properties during tendon development. J. Cell. Biochem..

[53] Riley G.P., Harrall R.L., Cawston T.E., Hazleman B.L., Mackie E.J. (1996). Tenascin-C and human tendon degeneration. Am. J. Pathol..

[54] Gaut L., Duprez D. (2016). Tendon development and diseases. Wiley Interdiscip. Rev. Dev. Biol..

[55] Derynck R., Budi E.H. (2019). Specificity, versatility, and control of TGF-b family signaling. Sci. Signal..

[56] De Mos M., Van El B., Degroot J., Jahr H., Van Schie H.T.M., Van Arkel E.R., Tol H., Heijboer R., Van Osch G.J.V.M., Verhaar J.A.N. (2007). Achilles tendinosis: Changes in biochemical composition and collagen turnover rate. Am. J. Sports Med..

[57] Riley G.P., Curry V., DeGroot J., Van El B., Verzijl N., Hazleman B.L., Bank R.A. (2002). Matrix metalloproteinase activities and their relationship with collagen remodelling in tendon pathology. Matrix Biol..

[58] Jabłońska-Trypuć A., Matejczyk M., Rosochacki S. (2016). Matrix metalloproteinases (MMPs), the main extracellular matrix (ECM) enzymes in collagen degradation, as a target for anticancer drugs. J. Enzyme Inhib. Med. Chem..

[59] Kjær M. (2004). Role of Extracellular Matrix in Adaptation of Tendon and Skeletal Muscle to Mechanical Loading. Physiol. Rev..

[60] Wiśniewski J.R., Zougman A., Nagaraj N., Mann M. (2009). Universal sample preparation method for proteome analysis. Nat. Methods.

[61] Gerarduzzi C., Hartmann U., Leask A., Drobetsky E. (2020). The matrix revolution: Matricellular proteins and restructuring of the cancer microenvironment. Cancer Res..

[62] Allamand V., Briñas L., Richard P., Stojkovic T., Quijano-Roy S., Bonne G. (2011). ColVI myopathies: Where do we stand, where do we go?. Skelet. Muscle.

[63] Marzeda A.M., Midwood K.S. (2018). Internal Affairs: Tenascin-C as a Clinically Relevant, Endogenous Driver of Innate Immunity. J. Histochem. Cytochem..

[64] Mattey D.L., Dawes P.T., Nixon N.B., Slater H. (1997). Transforming growth factor β1 and interleukin 4 induced α smooth muscle actin expression and myofibroblast-like differentiation in human synovial fibroblasts in vitro: Modulation by basic fibroblast growth factor. Ann. Rheum. Dis..

[65] Wunderli S.L., Blache U., Snedeker J.G. (2020). Tendon explant models for physiologically relevant in vitro study of tissue biology—A perspective. Connect. Tissue Res..

[66] Garcia-Melchor E., Cafaro G., MacDonald L., Crowe L.A.N., Sood S., McLean M., Fazzi U.G., McInnes I.B., Akbar M., Millar N.L. (2021). Novel self-amplificatory loop between T cells and tenocytes as a driver of chronicity in tendon disease. Ann. Rheum. Dis..

[67] Arvind V., Huang A.H. (2021). Reparative and Maladaptive Inflammation in Tendon Healing. Front. Bioeng. Biotechnol..

[68] Courneya J.P., Luzina I.G., Zeller C.B., Rasmussen J.F., Bocharov A., Schon L.C., Atamas S.P. (2010). Interleukins 4 and 13 modulate gene expression and promote proliferation of primary human tenocytes. Fibrogenes. Tissue Repair.

[69] Muller L., Hong C.S., Stolz D.B., Watkins S.C., Whiteside T.L. (2014). Isolation of biologically-active exosomes from human plasma. J. Immunol. Methods.

[70] Gonçalves A.I., Vinhas A., Rodrigues M.T., Gomes M.E. (2021). The impact of cryopreservation in signature markers and immunomodulatory profile of tendon and ligament derived cells. J. Cell. Physiol..

[71] Costa-Almeida R., Calejo I., Altieri R., Domingues R.M.A., Giordano E., Reis R.L., Gomes M.E. (2019). Exploring platelet lysate hydrogel-coated suture threads as biofunctional composite living fibers for cell delivery in tissue repair Biomedical Materials (Bristol).

[72] Babo P., Santo V.E., Duarte A.R.C., Correia C., Costa M.H.G., Mano J.F., Reis R.L., Gomes M.E. (2014). Platelet lysate membranes as new autologous templates for tissue engineering applications. Inflamm. Regen..

[73] Taylor S.C., Nadeau K., Abbasi M., Lachance C., Nguyen M., Fenrich J. (2019). The ultimate qPCR experiment: Producing publication quality, reproducible data the first time. Trends Biotechnol..

[74] Kulak N.A., Pichler G., Paron I., Nagaraj N., Mann M. (2014). Minimal, encapsulated proteomic-sample processing applied to copy-number estimation in eukaryotic cells. Nat. Methods.

[75] Hughes C.S., Moggridge S., Müller T., Sorensen P.H., Morin G.B., Krijgsveld J. (2019). Single-pot, solid-phase-enhanced sample preparation for proteomics experiments. Nat. Protoc..

