# Supplementary material for: Therapeutic Effects of Platelet-Derived Extracellular Vesicles in a Bioengineered Tendon Disease Model
Source: Int J Mol Sci. 2022 Mar 9;23(6):2948. doi: 10.3390/ijms23062948 (PMC8954460; doi:10.3390/ijms23062948)
Supplement: Supplementary file 1 [file ijms-23-02948-s001.zip › ijms-1604691-supplementary.pdf]

Supplementary Materials

Therapeutic Effects of Platelet-Derived Extracellular Vesicles in a Bioengineered Tendon Disease Model

Ana L. Graça [1,2], Rui M. A. Domingues [1,2], Isabel Calejo [1,2], Manuel Gómez-Florit [1,2,*,†] and Manuela E. Gomes [1,2,*]

[1] 3B's Research Group, I3Bs—Research Institute on Biomaterials, Biodegradables and Biomimetics, University of Minho, Headquarters of the European Institute of Excellence on Tissue Engineering and Regenerative Medicine, AvePark, Parque de Ciência e Tecnologia, Zona Industrial da Gandra, 4805-017 Barco, Guimarães, Portugal; ana.graca@i3bs.uminho.pt (A.L.G.); rui.domingues@i3bs.uminho.pt (R.M.A.D.); isabel.calejo@i3bs.uminho.pt (I.C.)
[2] ICVS/3B's–PT Government Associate Laboratory, 4805-017 Barco, Guimarães, Portugal
* Correspondence: mgflorit@i3bs.uminho.pt or manuel.gomezflorit@ssib.es (M.G.-F.); megomes@i3bs.uminho.pt (M.E.G.)
† Present address: Health Research Institute of the Balearic Islands (IdISBa), 07010 Palma, Spain.

Table S1. Quantitative extracellular matrix (ECM) proteomic analysis of isotropic fibers system encapsulating human tendon derived stem cells (hTDCs) supplemented with small extracellular vesicles (sEVs) and medium extracellular vesicles (mEVs). Protein description and accession numbers and functional classification are relative to *Homo sapiens* database from UniProt and PANTHER. The abundance percentage was obtained by dividing each protein abundance by the total abundance of the proteins identified in each condition.

Protein Description	Accession Number	Gene	Functional Classification	% Abundance		
				CTR	sEVs	mEVs

Basement membrane-specific heparan sulfate proteoglycan core protein	P98160	*HSPG2*	Basement membrane	0,053807746	0,01083986	0,017234883
Collagen alpha-1(VIII) chain	P27658	*COL8A1*	Basement membrane	0,003341779	-	0,009268878
Laminin subunit beta-1	P07942	*LAMB1*	Basement membrane	-	-	0,000369142
Latent-transforming growth factor beta-binding protein 2	Q14767	*LTBP2*	ECM Regulator	0,001618307	-	0,002202978
Latent-transforming growth factor beta-binding protein 1	Q14766	*LTBP1*	ECM Regulator	-	-	0,005527773
Collagen alpha-1(XII) chain	Q99715	*COL12A1*	FACIT Collagen	6,305374193	3,982406247	4,220302246
Collagen alpha-1(XIV) chain	Q05707	*COL14A1*	FACIT Collagen	0,940861499	0,909167793	0,838757036
Collagen alpha-1(III) chain	P02461	*COL3A1*	Fibrillar Collagen	0,027018423	0,012312724	0,024810318
Collagen alpha-1(V) chain	P20908	*COL5A1*	Fibrillar Collagen	0,097884033	0,054921704	0,033001446
Collagen alpha-1(I) chain	P02452	*COL1A1*	Fibrillar Collagen	0,555595133	0,312222078	0,44552708
Collagen alpha-3(V) chain	P25940	*COL5A3*	Fibrillar Collagen	0,004440607	0,00193585	-
Collagen alpha-1(II) chain	P02458	*COL2A1*	Fibrillar Collagen	0,096939344	-	0,063007013
Collagen alpha-2(V) chain	P05997	*COL5A2*	Fibrillar Collagen	0,063623519	-	0,065633662
Collagen alpha-2(I) chain	P08123	*COL1A2*	Fibrillar Collagen	0,092063075	-	-
Collagen alpha-1(XI) chain	P12107	*COL11A1*	Fibrillar Collagen	0,000783759	-	-
Prolyl 3-hydroxylase 3	Q8IVL6	*P3H3*	Matricellular	0,000770391	0,002226676	0,00646479
Prolyl 3-hydroxylase 1	Q32P28	*P3H1*	Matricellular	0,02021064	0,023614136	0,051213968

Growth arrest-specific protein 6	Q14393	*GAS6*	Matricellular	-	0,005423207	0,00465445
Fibulin-2	P98095	*FBNL2*	Matricellular	0,015855969	0,002137431	0,008687542
Tenascin	P24821	*TNC*	Matricellular	7,84977546	3,254504551	5,86808444
Galectin-3	P17931	*LGALS3*	Matricellular	0,000244547	0,001269397	-
Collagen alpha-3(VI) chain	P12111	*COL6A3*	Matricellular	74,17323372	80,47200237	78,01900631
Collagen alpha-2(VI) chain	P12110	*COL6A2*	Matricellular	9,647925189	10,93211187	10,26892455
Galectin-1	P09382	*LGALS1*	Matricellular	0,006345137	0,011410259	0,016989542
Matrilin-2	O00339	*MATN2*	Matricellular	0,006469426	0,001798267	0,001521885
Fibrillin-1	P35555	*FBN1*	Structural ECM	0,035818106	0,009695571	0,028810066

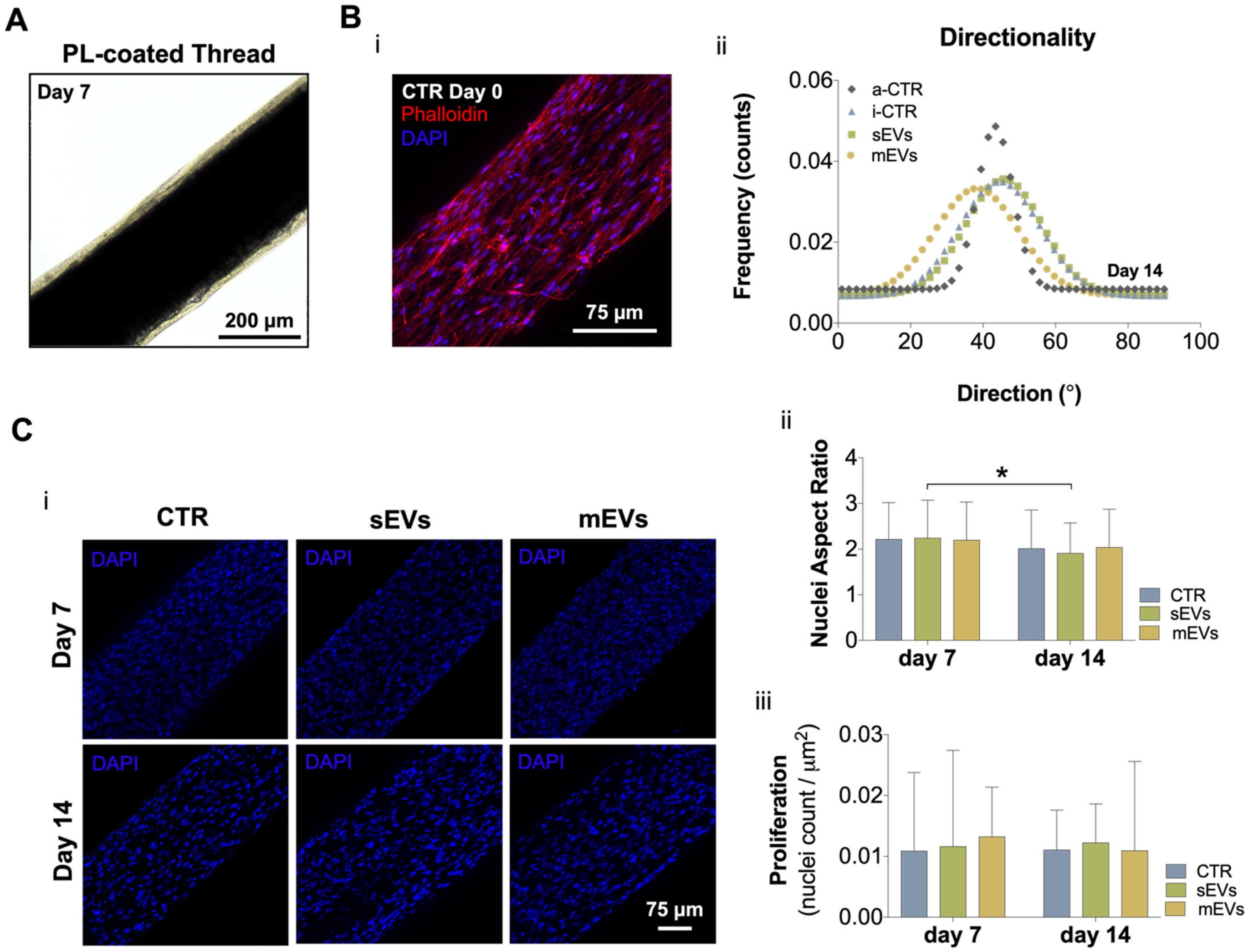

Supplementary Figure S1. (A) Platelet lysate (PL) coating retraction at day 7. Scale bar: 200 µm. **(B)** (i) Confocal images of F-actin filaments (DAPI, blue; Phalloidin, red) of encapsulated human tendon derived stem cells (hTDCs) before the extracellular vesicles (EVs) supplementation. Scale bar: 75 µm. (ii) Directionality analysis of F-actin filaments after 14 days. a-CTR: anisotropic control; i-CTR: isotropic control. **(C)** (i) Confocal images of hTDCs after 7 and 14 days of culture with EVs (DAPI, blue). Scale bar: 75 µm. (ii) Nuclei aspect ratio of hTDCs after 7 and 14 days of culture. Statistical differences: * $p < 0.05$. Data are presented as mean ± standard deviation (SD; n = 3). (iii) Cells proliferation after 7 and 14 days of culture (n = 3).

Table S2. Unique and shared proteins identified in the control samples and in the samples stimulated with small extracellular vesicles (sEVs) and medium extracellular vesicles (mEVs) using Venny 2.1.

Protein Description	Accession Number	Gene
**Common in CTR, sEVs and mEVs**		
Collagen alpha-3(VI) chain	P12111	*COL6A3*
Collagen alpha-2(VI) chain	P12110	*COL6A2*

Tenascin	P24821	*TNC*
Collagen alpha-1(XII) chain	Q99715	*COL12A1*
Collagen alpha-1(XIV) chain	Q05707	*COL14A1*
Collagen alpha-1(I) chain	P02452	*COL1A1*
Collagen alpha-1(V) chain	P20908	*COL5A1*
Basement membrane-specific heparan sulfate proteoglycan core protein	P98160	*HSPG2*
Fibrillin-1	P35555	*FBN1*
Collagen alpha-1(III) chain	P02461	*COL3A1*
Prolyl 3-hydroxylase 1	Q32P28	*P3H1*
Fibulin-2	P98095	*FBNL2*
Matrilin-2	O00339	*MATN2*
Galectin-1	P09382	*LGALS1*
Prolyl 3-hydroxylase 3	Q8IVL6	*P3H3*
**Common in CTR and sEVs**		
Collagen alpha-3(V) chain	P25940	*COL5A3*
Galectin-3	P17931	*LGALS3*
**Common in CTR and mEVs**		
Collagen alpha-1(II) chain	P02458	*COL2A1*
Collagen alpha-2(V) chain	P05997	*COL5A2*
Collagen alpha-1(VIII) chain	P27658	*COL8A1*
Latent-transforming growth factor beta-binding protein 2	Q14767	*LTBP2*
**Common in sEVs and mEVs**		
Growth arrest-specific protein 6	Q14393	*GAS6*
**Exclusive in CTR**		
Collagen alpha-2(I) chain	P08123	*COL1A2*
Collagen alpha-1(XI) chain	P12107	*COL11A1*
**Exclusive in mEVs**		

Latent-transforming growth factor beta-binding protein 1	Q14766	*LTBP1*
Laminin subunit beta-1	P07942	*LAMB1*

Table S3. RT-PCR primers used.

Gene	Primer Sequence	Accession Number
GAPDH	F: TGCACCACCAACTGCTTAGC<br>R: GGCATGGACTGTGGTCATGAG	NM_33197
GUSB	F: GATGTAGGTGGTGGGTGTCG<br>R: GCTCCGAATCACTATCGCCA	NM_000181.4
MKX	F: TCGCACAGACACTCTGGAAAA<br>R: TGTTAAGGCCATAGCTGCGT	NM_173576.2
SCX	F: AGAACACCCAGCCCAAACAGAT<br>R: TCGCGGTCCTTGCTCAACTTT	NM_001080514.2
TNC	F: ACTGCCAAGTTCACAACAGACC<br>R: CCCACAATGACTTCCTTGACTG	NM_002160.3
TNMD	F: CCGCGTCTGTGAACCTTTAC<br>R: CACCCACCAGTTACAAGGCA	NM_022144.2
COL3A1	F: TTGGCATGGTTCTGGCTTCC<br>R: GCTGGCTACTTCTCGTG	NM_000090.3
DCN	F: CAGCATTCCTCAAGGTCTTCCT<br>R: GAGAGCCATTGTCAACAGCA	NM_001920.3
COL1A1	F: GCCAAGACGAAGACATCCCA<br>R: GGCAGTTCTTGGTCTCGTCA	NM_000088.4
IL4	F: GCACCGAGTTGACCGTAACA<br>R: AGGAATTCAAGCCCGCCAG	NM_000589.3
IL6	F: AGGAGACTTGCCTGGTGAAA<br>R: GCATTTGTGGTTGGGTCAG	NM_000600.4

IL8	F: GGTGCAGTTTTGCCAAGGAG<br>R: TTCCTTGGGGTCCAGACAGA	NM_001354840.1
ACTA2	F: TAAGACGGGAATCCTGTGAAGC<br>R: TGTCCCATTCCCACCATCAC	NM_001141945.1
RUNX2	F: TTCCAGACCAGCAGCACTC<br>R: CAGCGTCAACACCATCATTC	NM_001024630
SOX9	F: TTCATGAAGATGACCGACGC<br>R: GTCCAGTCGTAGCCCTTGAG	NM_000346.3

Materials and Methods S1. Fiber's 3D Holder Fabrication

AutoCAD (version 2019) was used to design the three-dimensional (3D) polylactic acid (PLA, Beeverycreative, Portugal) support with an inner and outer diameter of 3.0 and 3.4 cm, respectively, to hold four threads with an estimated length of 2.5 cm. Also, a four channels support (depth – 0.4 cm, diameter 0.2 cm and height – 0.5 cm) was designed to coat individually each fiber. 3D holders were printed by using B2X300 printer (Beeverycreative, Portugal).
